# Feasibility of *lactiplantibacillus plantarum* postbiotics production in challenging media by different techniques

**DOI:** 10.1186/s13568-024-01704-5

**Published:** 2024-04-26

**Authors:** Mahsa Khakpour, Mohammad Mohsenzadeh, Amir Salari

**Affiliations:** Department of Food Hygiene and Aquaculture, Faculty of Veterinary Medicine, Ferdowsi University of Mashhad, Mashhad, Iran

**Keywords:** Postbiotics, *Lactiplantibacillus plantarum*, Antibacterial, Antifungal, Whey

## Abstract

The postbiotic derived from *Lactiplantibacillus plantarum* bacteria was produced in three culture media: milk, MRS, and whey, and its antibacterial and antifungal properties were evaluated. To investigate the production efficiency of postbiotics, three methods, heating, sonication and centrifugation, were utilized to prepare postbiotics in MRS broth culture medium. The antibacterial potency of the postbiotic was evaluated using the agar well-diffusion method, and MIC and MBC tests were conducted for different treatments. The results of the study showed that the postbiotic prepared in food environments such as milk and cheese whey can have antibacterial and antifungal properties similar to the postbiotic prepared in the MRS culture medium. However, it is possible to enrich food matrices such as milk and cheese whey and make further adjustments in terms of pH settings. Additionally, the thermal process was able to create a nanoscale postbiotic, which is a significant achievement for the application of postbiotics in the food and pharmaceutical industries. The future outlook of postbiotics clearly indicates that the emergence of this generation of probiotics can have an attractive and functional position in the food and pharmaceutical industries. Therefore, future research focusing on this subject will contribute to the development of this generation of postbiotics.

## Introduction

The emergence of the postbiotic concept is an end to the concerns among researchers regarding the limitations associated with the use of probiotics in food. Moreover, it signifies the advent of a new era in the food industry and the development of biological preservatives. While this concept has been several years old, its recent exploration has unveiled new dimensions (Sharafi et al. 2023). Fermented products have been consumed by humans for many years. Through the processing of foods such as dairy products, sourdough, and fermented meats, the cell walls of beneficial microorganisms are broken down, allowing the resulting metabolites to enter the food (Leyva Salas et al. 2017). However, recent studies are focused on engineer this implied process it to enable the large-scale production of metabolites derived from beneficial food-grade microorganisms. These metabolites hold promise for a wide range of applications in nutrition, healthcare, and various industrial purposes (Udayakumar et al. 2022). These postbiotics are the by-products of beneficial microorganisms, often produced by lactic acid bacteria (LAB). They consist of various intracellular and extracellular compounds, including vitamins, antimicrobial proteins, bioactive peptides, and bacterial biosurfactants (Rezaei, Nickfar Rezaei et al. 2023). In contrast to probiotics, where viability is a critical factor in generating health benefits, non-viable postbiotics can still have a positive impact on health due to the presence of significant cellular components and metabolites (Rezaei, Khanzadi and Salari Rezaei et al. 2021a, b; Rezaei, Salari and Khanzadi Rezaei et al. 2021a, b). Compared to live bacterial cells, postbiotics offer several valuable advantages in terms of food use. These include longer shelf life, structural safety, no transfer of antibiotic resistance, absence of biogenic amine (BA) production, ease of use and storage, stability across a wide range of pH and temperature, and broad-spectrum antimicrobial activity against bacteria and fungi (Moradi et al. 2020). Recent studies have demonstrated the effectiveness of postbiotics as antimicrobial compounds in preserving packaged meat, sausages, and chicken fillets. Additionally, the use of postbiotics as biological preservatives in various cheeses has shown positive effects (Sharafi et al. 2022). Another noteworthy characteristic of postbiotics is their anti-biofilm properties against pathogenic bacteria, which can have significant implications for the food industry (Rezaei et al. 2022). In the dairy industry, the utilization of postbiotics has yielded positive outcomes, including the prevention of undesirable sensory changes in dairy products, such as excessive acidity that may occur during yogurt and other dairy product fermentations (Rezaei et al. 2023). Moreover, due to their abundance in extracellular polysaccharides, postbiotics contribute to improved stability and mouthfeel by influencing the texture of the product. Previous studies have indicated that postbiotics derived from the Saccharomyces genus can impact the cheese ripening process through the presence of polyamines like putrescine and spermine, which possess pectinolytic properties. Multiple studies have consistently demonstrated the antimicrobial effects of various postbiotics against foodborne pathogens, including *L. monocytogenes* and *E. coli. Listeria monocytogenes* has been a main target in most postbiotic studies due to the importance of this bacterium in food hygiene. The results of previous studies showed that postbiotic prepared from *L. plantarum* NRRL B-4496 was effective in reducing the population of *S. Typhimurium* and *L. monocytogenes* by 3.74 log CFU/g and 2.3 log CFU/respectively during 14 days in refrigerator storage. (Lin and Pan 2019; da Costa et al. 2021; Sharafi et al. 2023). However, most studies on postbiotics have been conducted based on probiotic growth in culture media. The yellow-brown color of postbiotics formed in MRS broth limits their applicability in various food products. The preparation of postbiotics using culture media can result in a red to brown color in products such as dairy items that are typically expected to have a white appearance (Sharafi et al. 2023). For the successful commercialization and integration of postbiotics in food settings, there is a requirement for affordable, safe, and food-compatible culture media. In this context, milk regards as an ideal environment due to its rich composition of micronutrients and essential materials necessary for cultivation. Specifically, whey can be regarded as an economically advantageous medium for this purpose. The centrifuge technique has been widely used in laboratory and research studies to disrupt the cell wall and extract the cell free supernatant. However, for the successful commercialization of postbiotics, it is essential to develop methods that not only offer improved efficiency but also enhanced effectiveness. In this study, the feasibility of producing nano postbiotics from *La. plantarum* was investigated using different methods such as: heating, sonication and centrifuge in different media namely MRS culture media, milk, and whey. Considering that the preparation of postbiotics in the laboratory culture media creates application limitations, by examining other food culture environments for the extraction of postbiotics, a new approach can be created in the food industry and the prospect of using natural preservatives in the food industry can be developed.

## Materials and methods

### Microbial strains and cultures preparation

The lyophilized LAB strain (*La. plantarum* PTCC1745) was activated and transferred into De Man, Rogosa, and Sharpe (MRS) broth or agar (Oxoid, Milan, Italy). *Listeria monocytogenes* (ATCC 7644), *Staphylococcus aureus* (ATCC 25,923), *Escherichia coli* (NCTC12900) and *Salmonella typhi morium* (ATCC 14,028) were cultured in Tryptic Soy broth (TSB, Oxoid). Fresh microbial suspensions of *La. plantarum*, the pathogenic bacteria, and spoilage mold strains were prepared by sub-culturing in De Mann, Rogosa, and Sharpe (MRS; Ibresco, Karaj, Iran) broth, Brain Heart Infusion (BHI; Ibresco, Iran) broth, and Potato Dextrose (PDA; Ibresco, Iran) agar, respectively. Aspergillus niger (PTCC 5010) and Aspergillus flavus (PTCC 5004) spores were used as target molds. These suspensions were standardized using a visible-ultraviolet spectrophotometer (Mecasys Co., Ltd., Daejeon, Korea) at 600 nm.

### Experimental media for *La. plantarum*

The experimental media used in this study were MRS broth, cheese whey, and low-fat UHT milk with a 1.5% fat content. Cheese whey was provided by the Pegah dairy factory located in Mashhad, Iran, and UHT milk was purchased from a local market. The whey was prepared by reducing the pH to 4.5 using 5 N HCl (Merck, Darmstadt, Germany), denaturing the proteins through autoclaving at 121 °C for 15 min, and removing the precipitates by centrifugation (2360×g for 15 min). (Sharafi et al. 2022).

### Growth kinetics of *La. plantarum* in selected cultures

A 2% overnight anaerobic culture in MRS broth was used to inoculate the experimental media. The cultures were incubated anaerobically at 37 °C for 36 h. The logarithmic value of bacterial cell concentration for each sample at 24 h was determined through dilution and pour plate counting. The samples were incubated in MRS agar at 37 °C for 48 h under anaerobic conditions.

### Preparation of postbiotics solutions

*La. plantarum* was cultured in MRS broth, cheese whey, and low-fat UHT milk and incubated at 37 ± 1 °C for 48 h. The bacterial suspensions obtained from these three media were divided into three separate groups. The first group was heat-killed in a water bath at 95 °C for 5 min. The second group was sonicated for 5 min, and the last group remained intact. Then, all three groups were centrifuged at 4200×g for 10 min at 4 °C and subsequently filter-sterilized through a 0.45-µm-pore filter. The supernatant was harvested and stored at 4 °C (İncili et al. 2022).

### Minimum inhibitory (MIC) and minimum bactericidal (MBC) concentrations

The micro-dilution assay method in broth was used to determine the minimum inhibitory concentrations (MICs) of postbiotics derived from *La. plantarum* cultured in MRS broth, cheese whey, and low-fat UHT milk. (Lin and Pan 2019). Seventy-five microliters of a diluted culture of pathogenic bacteria (10^6^ CFU/mL) were added to each well of a 96-well polystyrene flat-bottomed microtitre plate. The postbiotics were serially diluted to concentrations of 10,000, 8,000, 6,000, 5,000, 4,000, 3,000, 2,500, 2,000, 1,000, 500, and 250 µg/mL. Subsequently, 75 µL of each dilution was added to the respective wells. The treatment and control wells, which contained only bacterial suspensions, were then incubated at 37 °C for 18–20 h. The minimum inhibitory concentration (MIC) was determined as the lowest concentration of CFS (cell-free supernatant) that showed no observable growth of the tested organisms upon macroscopic examination. The MIC value was reported in micrograms per milliliter (µg/mL). Furthermore, the concentrations that completely suppressed visible growth of the bacterial pathogens were determined. Next, 50 µL of each culture broth was plated onto agar plates and incubated at 37 °C for 24 h. The minimum bactericidal concentration (MBC) was determined as the concentration at which no bacterial colonies were observed to develop on the agar surface (Bajpai et al. 2016).

### Antibacterial activity of postbiotics

The antibacterial activity of the postbiotics prepared from *La. plantarum* was determined using the agar well-diffusion method (Koohestani et al. 2018). Lawn cultures of pathogenic strains (6 log10 CFU/mL) were prepared on Mueller Hinton (MH) agar (Ibresco, Iran) and allowed to stand for 5 min. Wells were then created in the agar using a sterile filter pipette tip, and 100 µL of the prepared *La. plantarum* postbiotic was added to each well against each of the tested pathogens. Negative control was prepared using the same medium (sterile MRS medium) that was used to dissolve the samples. The plates were incubated at 37 ± 1 °C for 24 h. The antibacterial activity was assessed by measuring the zones of inhibition, which included the diameter of the well (6 mm), against the tested bacteria. The experiments were conducted in triplicate.

### Antifungal activity of postbiotics

The antifungal activity was measured using the agar well-diffusion method (Poornachandra Rao et al. 2019). *Aspergillus niger* and *Aspergillus flavus* spores were used as target molds. A total of 10 µL of the conidial suspension (10^6^ spores/mL) was evenly spread on PDA plates, and allowed to settle for 5 min. Wells with a diameter of 6 mm were then created in the agar. Each well was filled with 100 µL of postbiotics derived from *La. plantarum*. The plates were incubated at 30 °C for 48 h, and clear inhibition zones were observed. As a control, sterile MRS broth was used. The experiments were conducted in triplicate.

### Determination of particle size

Average particle size was measured using dynamic light scattering (DLS) at a fixed angle of 135°. The diameter of the particles was measured based on the function of the intensity of the scattered light assuming that they are spherical. All experiments were performed at 25 ± 2 °C in triplicate. Size Particles were reported by intensity.

### Statistical analysis

The data study was analyzed by analysis of variance (ANOVA (with GraphPad Prism version 5.0 for Windows. Duncan’s test was used to assess significant differences (*p* < 0.05) between the groups. All treatments were conducted in three plicate.

## Results

**Fig. 1:**
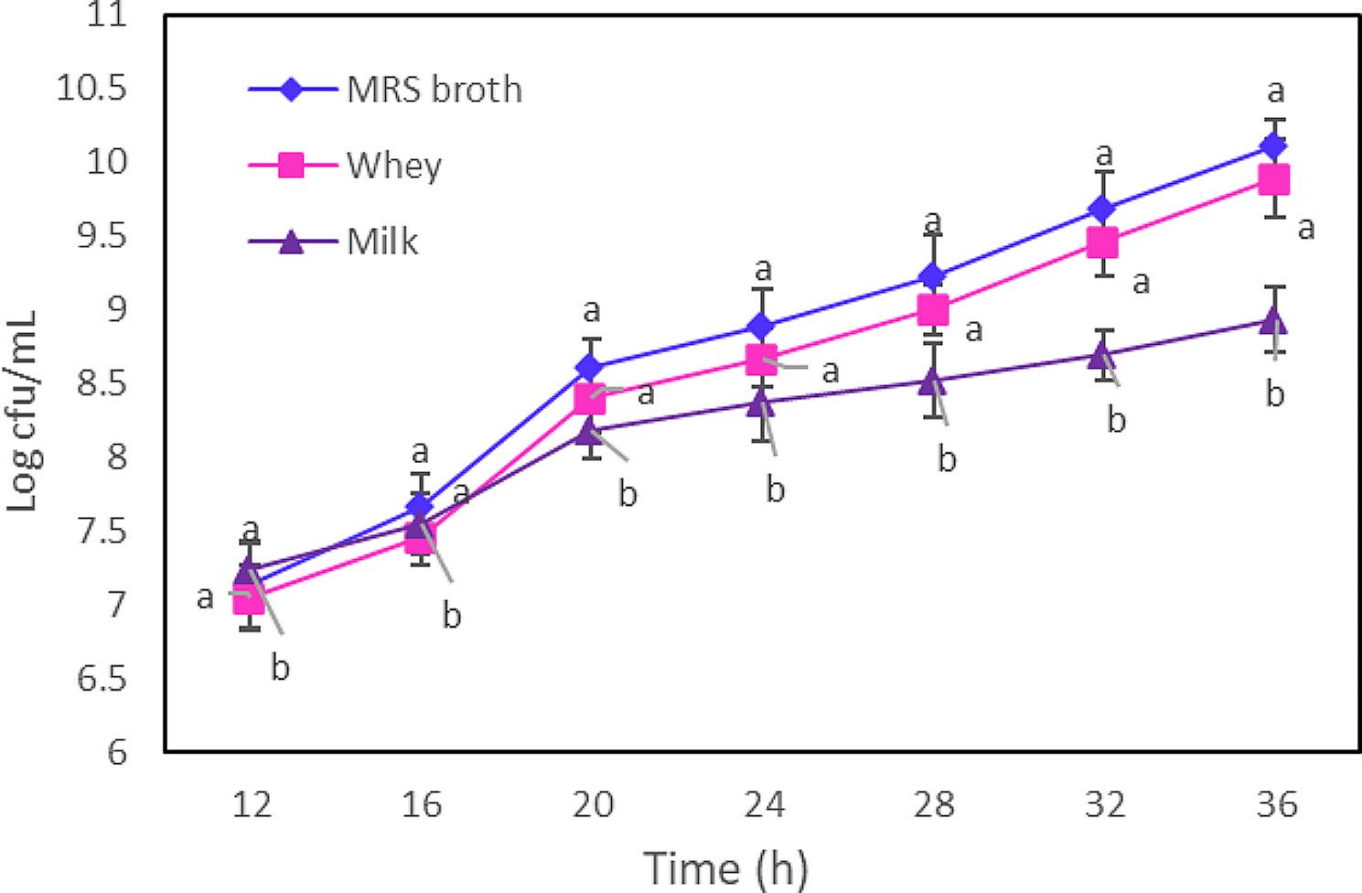
Growth curves (log CFU /mL) of *La. plantarum* in MRS broth, whey, and milk

The results revealed that the growth of probiotic bacteria in the MRS broth medium was greater than that in the milk culture medium. However, this difference was not statistically significant when compared to the growth of bacteria in the whey medium (Fig. 1).

### Antibacterial and antifungal activity of postbiotics

The postbiotic derived from *La. plantarum* exhibited antibacterial and antifungal activity, but its effectiveness varied across different environments, including milk, MRS broth, and whey (Table 1). Comparing the results, it was found that the postbiotic prepared in the three-culture media (whey, MRS broth, and milk) demonstrated the highest antimicrobial efficacy against the food pathogen *L. monocytogenes*. However, the inhibitory effect was greater in the postbiotic prepared with MRS broth compared to whey and milk culture media. As shown in Table 1, the mean diameter of the inhibition zone for the postbiotic prepared in MRS broth (17.0 ± 0.0 mm) and whey culture medium (15.5 ± 1.2 mm) was larger against *L. monocytogenes* compared to milk (12.8 ± 0.4 mm).

**Table 1:** Antibacterial activity of *La. Plantarum* against some pathogens (mm) in different media

Pathogen	Postbiotic prepared from La. plantarum in different environments		
	MRS	Whey	Milk
*Staphylococcus aureus*	13.5 ± 1.2	11 ± 0.4	12.0 ± 0.0
*Listeria monocytogenes*	17.0 ± 0.0	15.5 ± 1.2	12.8 ± 0.4
*Escherichia coli*	15.2 ± 1.1	12.0 ± 0.0	11.3 ± 0.6
*Salmonella typhi morium*	12.1 ± 0.6	13 ± 0.0	9.5 ± 0.0
*Asp. niger*	11.8 ± 0.5	9.5 ± 0.6	*ND
*Asp. Flavous*	10.2 ± 0.3	8.5 ± 0.59	ND

*ND: Not detected

According to the results presented in Table 2, there is a notable distinction among various techniques employed for the extraction of postbiotics. The heating technique exhibits a superior efficacy in extracting antimicrobial metabolites. However, no significant difference was observed between the centrifugation and sonication techniques. Similarly, just as there are variations in the antibacterial properties of postbiotics in different environments, the techniques employed also displayed varying effects, with the most pronounced impact observed on *L. monocytogenes* and *E. coli*.

**Table 2:** Antibacterial activity of *La. Plantarum* against some pathogens (mm) in different media

Pathogen	Postbiotic prepared from La. plantarum in different methods		
	Centrifugation	Sonicate	Heat
*Staphylococcus aureus*	13.5 ± 1	12.3 ± 0.5	15.7 ± 0.5
*Listeria monocytogenes*	17.0 ± 0.0	17.0 ± 0.0	18.8 ± 0.9
*Escherichia coli*	15.2 ± 1	14.8 ± 0.4	17.3 ± 0.7
*Salmonella typhi morium*	12.1 ± 0.6	12.5 ± 0.5	14.5 ± 1
*Asp. niger*	11.8 ± 0.5	11.3 ± 0.5	13.8 ± 0.2
*Asp. Flavous*	10.2 ± 0.3	10.2 ± 0.7	13.0 ± 0.6

### Minimum inhibitory (MIC) and minimum bactericidal (MBC) concentrations

**Fig. 2:**
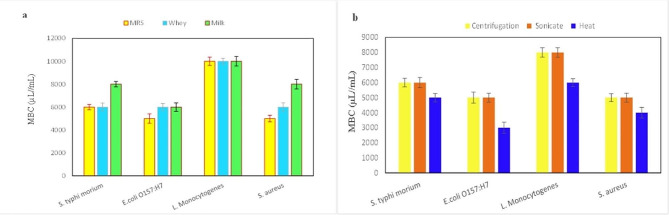
Minimum Bactericidal Concentrations (MBCs) of the *La. plantarum* postbiotic preparation in different media (a) and different methods (b) on food pathogens

**Fig. 3:**
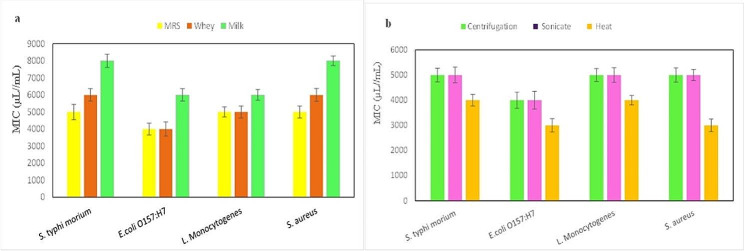
Minimum inhibitory concentrations (MICs) of the *La. plantarum* postbiotic preparation in different media (a) and different methods (b) on food pathogens

Food pathogens were exposed to different concentrations of postbiotic derived from *La. plantarum*, and each pathogen exhibited sensitivity at varying concentrations. As depicted in Figs. 2 and 3, the postbiotic prepared in the MRS culture medium, when compared to milk at a lower concentration, resulted in pathogen sensitivity. However, in the MBC test, the postbiotic prepared in the whey culture medium showed similar results to postbiotics prepared in the MRS environment in terms of *L. monocytogenes* and *S. typhi morium* bacteria, with no significant difference observed (*p* ≥ 0.05).

### Postbiotics-NPs

**Fig. 4:**
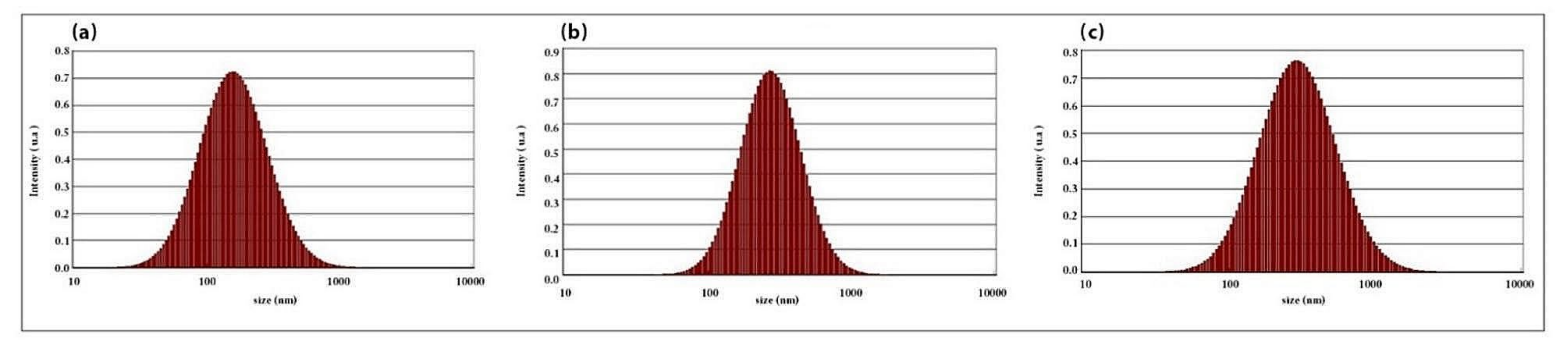
Comparison of postbiotic nanoparticle sizes in the various methods: (a): Heat, (b): Centrifugation, (C): Sonicate

As depicted in Fig. 4, the postbiotics obtained through different production processes exhibited a nanoparticle size. A comparison of the various production methods indicated that heat treatment yielded superior results in the production of nanoparticle postbiotics compared to sonication and centrifugation methods.

## Discussion

The composition of the culture medium used in the preparation of postbiotics had a significant impact on the chemical composition of the resulting postbiotics. The presence of high levels of glucose in the MRS broth medium resulted in increased levels of carboxylic acids in the final postbiotic product. (Sharafi et al. 2023). The use of MRS broth as a culture medium for postbiotic production has been common; however, it has certain limitations. One of these limitations is the development of a brown to yellow-brown color in the postbiotics prepared in MRS broth, which poses a challenge when utilizing them in dairy products. This color defect becomes more pronounced at higher concentrations and can impact the appearance and sensory quality (Sharafi et al. 2023). Considering the expensive nature of these cultures, it would be beneficial to explore cost-effective alternatives, particularly utilizing by-products and food waste, as viable resources for postbiotic production instead of relying solely on laboratory culture environments (İncili et al. 2022). Whey has been considered as an alternative medium for postbiotic production by researchers. Optimizing the pH of this culture medium and autoclaving it to denature proteins, along with the addition of supplements such as yeast extract and sugars, can result in the production of postbiotics with an appealing appearance that are compatible with various food products, particularly those derived from dairy sources. Sharfi et al. conducted a study on the effects of postbiotics prepared with whey on the shelf life of High-moisture mozzarella cheese. The results showed that the postbiotics exhibited a strong antibacterial effect, leading to a reduction of mesophiles and psychrophiles by 1.5-2 log CFU/g. Additionally, the microbial quality of the cheese was well preserved during storage. Milk also holds potential as a favorable culture environment. The presence of various supplements, such as glucose, yeast extracts, and other relevant compounds, can enhance the production of new metabolites, including bacteriocins and proteins with therapeutic and functional properties. Previous studies have demonstrated that the preparation of postbiotics using low-heat milk from different LAB preparations exhibits significant antifungal activity. (Garnier et al. 2019). Several factors contribute to the antibacterial effects of postbiotics. Compounds such as organic acids, hydrogen peroxide, bacteriocins, and antimicrobial peptides play significant roles in their antibacterial effects. One advantage of postbiotics is that their inhibitory activity remains unaffected by environmental factors, making them easy to use and maintain (El-Mokhtar et al. 2020). Recent studies have demonstrated that postbiotics can be a suitable strategy for inhibiting the growth of problematic pathogens (Chen et al. 2019). Interestingly, the antimicrobial activity of postbiotics is not limited to bacteria; they can also exhibit antifungal effects. The probiotic bacterium *La. plantarum* destroys fungal cell structures through the production of various metabolites, including lactic acid, fatty acids, phenolic compounds (such as plantarisin), and possesses a high ability to inhibit fungal growth. Additionally, amino acids, organic acids, and enzymes produced by *La. plantarum* are capable of binding to mycotoxins and neutralizing them (Fahim et al. 2021). Recent studies have shown that postbiotics obtained from Lactobacillus target a wide range of Gram-negative and Gram-positive bacteria, including S. aureus, *L. monocytogenes*, *S. typhimurium*, and *E. coli.* (Mani-López et al. 2022). The presence of lactic acid, acetic acid, ethanol, and H_2_O_2_ leads to significant antifungal activity against *P. corylophilum* after 7 days at 25 °C. In the Schmidt study, the cell-free supernatant (CFS) from *L. reuteri* R29, which contains reuterin, also exhibited antifungal activity against *A. niger* FST4.21 and *F. culmorum* TMW4.2043, inhibiting > 63% of their growth after 5 days (Schmidt et al. 2018). The presence of compounds such as peptone in the culture medium used for postbiotic production can enhance their antimicrobial properties. Various sources have been utilized for postbiotic production, including sweet whey (Silva et al. 2023), modified ultrafiltered whey (Sharafi et al. 2022), cheddar whey (Ünlü et al. 2016), hydrolyzed whey containing soy flour (da Silva Sabo et al. 2017) and low low-heat milk (Garnier et al. 2019). However, certain operational treatments are necessary, such as adjusting the pH to approximately 4.5 to 5 and autoclaving the culture to denature proteins for optimal postbiotic performance (Amiri et al. 2019). Thermal processes are commonly employed for bacterial postbiotic production. Nevertheless, different temperatures and times have been used across studies to prepare postbiotics (e.g., 121 °C for 15 min, 2 h at 60 °C, or 90–95 °C for 30 s). Thermal processes disrupt cell membranes and DNA, deactivate enzymes, and cause protein coagulation. While non-thermal approaches like sonication are not widely utilized, they offer greater efficiency and advantages over thermal treatments, including reduced energy consumption and the presence of specific metabolites in the postbiotic solution (Sharafi et al. 2023). Recently, studies have revealed that postbiotics derived from microorganisms exist in the form of nanoparticles, which encompass various genetic materials, small RNAs, short-chain lipids, and exopolysaccharides. The nanoparticle form of postbiotics not only enhances their antimicrobial activities but also contributes to their long-term stability (Salminen et al. 2021). These findings were further supported by the results of our present study. In the study conducted by Teame et al. (2020), the production of postbiotics from *Lactobacillus helveticus* PJ4 and *Lactobacillus brevis* DT24 demonstrated inhibition and suppression of *E. coli (*Teame et al. 2020). In another study by Liu et al., it was concluded that nanoparticle postbiotics containing cell wall degrading enzymes exhibited strong antibacterial properties, and the composition of nanoparticles effectively supported the functionality of postbiotics, particularly in the field of natural food preservation (Liu et al. 2018). Emerging evidence suggests that postbiotics-NPs, due to their particle size, hold potential for utilization as nutrients and drug delivery vehicles in the fields of functional food and biomedicine. Optimizing the production parameters of postbiotics is crucial and can yield numerous benefits, including cost-effectiveness and wider application of postbiotics in food preservation (Sharafi et al. 2023). The feasibility of postbiotic production utilizing food-grade culture medium was one of the approaches explored in this study. The findings of this study indicate that food industry waste, such as whey, can be effectively optimized and utilized as a natural cultivation environment for producing postbiotics, particularly in the dairy industry. An additional accomplishment of this study is the production of nanoparticle postbiotics through the heating process. The natural production of postbiotics in nanoparticle form presents an attractive and practical approach for their utilization in the food and pharmaceutical industries. Consequently, the production of postbiotics in food-compatible environments represents a novel perspective in the food industry that may pique the interest of researchers in the future. Furthermore, whey and milk can be regarded as potential fields for postbiotic production that align with food standards. However, various processes are required to optimize these environments in order to achieve optimal results in the production of postbiotics with significant antibacterial and antifungal properties.

## Data Availability

The corresponding author could provide all experimental data on a valid request.
